# Minor Proteins Involved in Neuronal Functions in Serum of Patients With Major Mental Disorders According to Proteomic Analysis

**DOI:** 10.31083/AP53136

**Published:** 2026-10-23

**Authors:** Liudmila Smirnova, Alexander Seregin, Evgeny Ermakov, Maria Zavialova, Elena Dmitrieva, Svetlana Novikova, German Simutkin, Elena Kornetova, Svetlana Ivanova

**Affiliations:** ^1^Laboratory of Molecular Psychiatry, Mental Health Research Institute, Tomsk National Research Medical Center of the Russian Academy of Sciences, 634014 Tomsk, Russia; ^2^Laboratory of Repair Enzymes, Institute of Chemical Biology and Fundamental Medicine, Siberian Branch of the Russian Academy of Sciences, 630090 Novosibirsk, Russia; ^3^Advanced Mass Spectrometry Core Facility, Skolkovo Institute of Science and Technology, 121205 Moscow, Russia; ^4^Laboratory of Systems Biology, Institute of Biomedical Chemistry, 119121 Moscow, Russia; ^5^Affective States Department, Mental Health Research Institute, Tomsk National Research Medical Center of the Russian Academy of Sciences, 634014 Tomsk, Russia; ^6^Endogenous Disorders Department, Mental Health Research Institute, Tomsk National Research Medical Center of the Russian Academy of Sciences, 634014 Tomsk, Russia; ^7^Psychiatry, Addictology and Psychotherapy Department, Siberian State Medical University, 634050 Tomsk, Russia

**Keywords:** schizophrenia, bipolar disorder, NMDAR1, anti-NMDAR antibodies, ANK3, ANKRD12, LRP2, dermcidin, synaptopathy, biomarkers

## Abstract

**Background:**

Schizophrenia (SCZ) and bipolar disorder (BD) share common clinical features, complicating the identification of specific peripheral biomarkers. The objective of this study was to identify biomarkers that specifically characterize different clinical forms of BD and SCZ.

**Methods:**

A comparative proteomic analysis of serum samples from patients with SCZ and BD, as well as healthy controls, was performed using untargeted mass spectrometry to identify differentially expressed proteins. In an expanded cohort, accounting for clinical heterogeneity, the levels of six minor proteins (N-methyl-D-aspartate receptor 1 (NMDAR1), ankyrin 3 (ANK3), ankyrin repeat domain-containing protein 12 (ANKRD12), low-density lipoprotein receptor-related protein 2 (LRP2), dermcidin (DCD), and anti-NMDAR antibodies) were quantified using enzyme-linked immunosorbent assay (ELISA) and targeted mass spectrometry (for NMDAR1).

**Results:**

Comparative serum proteomic analysis identified 440 unique proteins in healthy controls versus 221 in patients with SCZ, and 474 unique proteins in healthy controls versus 55 in patients with BD. Quantitative analysis revealed the following findings: (1) a significant increase in NMDAR1 protein levels in patients with BD experiencing a current depressive episode (BD-cDE); (2) decreased ANK3 levels in patients with BD-cDE and SCZ; (3) increased ANKRD12 protein levels in patients with SCZ characterized by predominant negative symptoms and in patients with BD-cDE; (4) decreased DCD levels in patients with SCZ in remission compared with those in the acute phase of the disease; and (5) significantly increased LRP2 levels in both BD and SCZ. Elevated anti-NMDAR antibody levels were more frequently observed in patients with BD, although absolute antibody levels were also increased in patients with acute-phase SCZ.

**Conclusions:**

These findings suggest a common mechanism of synaptopathy involving destabilization of the ANK3-NMDAR scaffold, accompanied by a compensatory neuroprotective response in both BD and SCZ. The identified alterations in the serum protein profile may serve as potential biomarkers for the differential diagnosis of these disorders as part of multimarker diagnostic panels.

## Main Points

1. Using panoramic mass spectrometry, differentially expressed proteins were identified in bipolar disorder (BD) and schizophrenia (SCZ) patients compared to healthy controls.

2. Quantitative analysis revealed significant changes in protein levels [N-methyl-D-aspartate receptor 1 (NMDAR1), ankyrin 3 (ANK3), ankyrin repeat domain-containing protein 12 (ANKRD12), low-density lipoprotein receptor-related protein 2 (LRP2), dermcidin (DCD)] in BD patients depending on the phase of the current episode and, to a lesser extent, in SCZ patients depending on the leading symptomatology.

3. The presence of NMDAR1 protein in serum along with high levels of NMDAR antibodies was more often detected in patients with BD.

4. Several changes were consistent, suggesting a common mechanism of “synaptopathy” associated with destabilisation of the ANK3-NMDAR scaffold in BD and SCZ.

## 1. Introduction

Schizophrenia (SCZ) and bipolar disorder (BD) are major mental disorders with complex genetic and pathophysiological underpinnings [1,2]. They often present overlapping symptoms and high comorbidity rates [3], suggesting shared neurobiological mechanisms [4,5]. BD and SCZ affect approximately 2.4% and 1% of the population, respectively, and are associated with a significant global burden, reduced quality of life, and high treatment costs [6].

The neurobiological basis of SCZ and BD remains poorly understood, complicating the search for pathogenesis-based biomarkers and novel treatment strategies. Genome-wide association studies have revealed associations with multiple genes, each with minimal individual contribution, and substantial genetic overlap between these disorders [5,7,8]. At the core of SCZ and BD pathophysiology are neurotransmitter disturbances leading to neural circuit dysfunction and brain dysconnectivity, although the triggers for these changes remain poorly understood [9,10,11]. Recent evidence has highlighted the role of altered glutamatergic and GABAergic neurotransmission, as well as changes in N-methyl-D-aspartate receptor (NMDAR) conductance [9,10,11], but reliable markers to assess these changes have not yet been identified. Additionally, neurotransmitter abnormalities in SCZ and BD are associated with mitochondrial dysfunction and oxidative stress [12,13]. Finally, numerous studies indicate a role for neuroinflammation linked to blood–brain barrier dysfunction in both disorders [14,15,16,17]. Consequently, pathophysiological brain processes may lead to the appearance of neuronal proteins in cerebrospinal fluid and blood, whose peripheral levels could reflect the extent of brain changes.

There is a growing need for peripheral markers that can support diagnosis and treatment outcome prediction (theranostics) in mental disorders. Modern high-throughput methods, particularly proteomic analysis, enable the identification of protein biomarkers associated with functional impairments involved in disease pathophysiology. However, most recent proteomic studies have primarily revealed changes in highly abundant serum proteins that are not specific to mental disorders [18,19,20]. The difficulty in detecting low-abundance proteins in serum stems primarily from the dynamic range of the mass spectrometer (where high-abundance proteins mask weaker signals) and the data acquisition modes. Therefore, the identification of neurospecific proteins of great interest for biological psychiatry depends on sample pre-processing (e.g., depletion of high-abundance proteins), highly sensitive equipment, and the optimal choice of sequence database search algorithms. The MASCOT search engine (Matrix Science) is one of the optimal algorithms for searching for proteins present in very low concentrations in patient serum [21].

Based on recent proteomic literature, our previous findings [22,23,24], and the data obtained in this study, several neurospecific proteins were selected for further investigation. Among these proteins were NMDAR subunits. Functional NMDAR is known to be a heterotetramer composed of combinations of NMDAR1, NMDAR2, and NMDAR3 subunits derived from three gene families [25]; the most common subunits are NMDAR1 (GluN1) and NMDAR2 (GluN2 (A–D)) [26]. Furthermore, variants of the genes encoding these subunits (rs7206256, rs11644461 for GRIN2A and rs7313149 for GRIN2B) were associated with early onset SCZ [27]. Dermcidin (DCD), primarily known for its antimicrobial activity [28], also exhibits a broad range of other biological functions related to immunomodulation in response to inflammation and oxidative stress. Notably, experimental studies have shown that the N-terminal peptide of DCD promotes neuronal survival under oxidative stress conditions [29]. Another protein, low-density lipoprotein receptor-related protein 2 (LRP2), or megalin, is a large multifunctional receptor involved in endocytosis and cellular signalling, and has been linked to SCZ pathogenesis [30]. Ankyrin repeat proteins are also of interest in the context of mental disorders [31]. Ankyrin repeat domain-containing protein 12 (ANKRD12), also known as ANCO-2, inhibits transcriptional activity by interacting with histone deacetylases (HDACs). Postmortem brain studies have confirmed dysregulation of HDAC1 and HDAC2 expression in SCZ and BD [32,33]. Our previous study showed an increase in ANKRD12 concentration with increasing BD duration [34]. Ankyrin 3 (ANK3, ankyrin-G) is an axonal cytoskeletal protein that regulates gamma-aminobutyric acid (GABA) receptor binding and is essential for retaining GABA_A_ receptors on the neuronal surface [35,36]. The *ANK3* gene is considered one of the key common genetic risk markers for BD and SCZ, which is confirmed by changes in *ANK3* expression in both pathologies [37].

Thus, the main objective of this study was to identify biomarkers that specifically characterise different clinical forms of BD and SCZ and to assess their potential utility in clinical practice, primarily for differential diagnosis.

## 2. Materials and Methods

### 2.1 Characteristics of Individuals Included in the Study

This study included 80 patients with SCZ with a median age of 32.0 [26.0; 43.0] years and a disease duration of 6.0 [2.0; 13.0] years; 51 patients with BD with a median age of 41.0 [26.0; 51.0] years and a disease duration of 8.0 [3.0; 18.0] years, and 40 healthy individuals aged 33.0 [28.0; 41.0] years. The nosological composition of the SCZ group included individuals with simple (F20.6, n = 24) and paranoid (F20.0, n = 56) SCZ. Paranoid SCZ with continuous course (F20.01) (n = 22); paranoid SCZ with episodic course and stable defect (F20.02) (n = 20); paranoid SCZ in a state of incomplete remission (F20.09) (n = 14). The BD group included individuals with a current mixed episode (F31.6, n = 29) and a current depressive episode of varying severity (n = 22), including the following: mild or moderate depression (F31.3, n = 14); severe depression without psychotic symptoms (F31.4, n = 5); severe depression with psychotic symptoms (F31.5, n = 3). No statistically significant correlations were found between the participants’ ages and the parameters studied (*p* > 0.05). The group of healthy individuals was recruited from students of the Siberian State Medical University and employees of Tomsk National Research Medical Center of the Russian Academy of Sciences (TNRMC) who underwent a medical examination and filled out a special questionnaire.

Patient groups were formed and clinically verified at the Department of Endogenous Disorders and the Department of Affective States of the Mental Health Research Institute (MHRI), Tomsk National Research Medical Center of the Russian Academy of Sciences (TNRMC). Diagnosis verification was conducted by qualified psychiatrists according to the International Classification of Diseases, 10th Revision (ICD-10) criteria.

In the first stage, comparative proteomic profiling of blood serum from the recruited groups was conducted: patients with SCZ (n = 16), BD (n = 15), and controls (n = 11). The group of SCZ patients was represented by the following clinical forms: simple SCZ (F20.6) (n = 4); paranoid SCZ with continuous course (F20.01) (n = 4); paranoid SCZ with episodic course and stable defect (F20.02) (n = 4); paranoid SCZ in a state of incomplete remission (F20.09) (n = 4). At this stage of the work, the general group of patients with SCZ was analysed, without taking into account clinical features. The median age of patients with SCZ was 30.8 [24.0; 47.5] years, and the median duration of the disease was 4.5 [0.5; 12.5] years. The BD group included patients with a current mixed episode (F31.6, n = 9) and depressive episodes of varying severity: mild or moderate depression (F31.3, n = 2), severe depression without psychotic symptoms (F31.4, n = 2) and severe depression with psychotic symptoms (F31.5, n = 2). The median age of patients with BD was 31.0 [25.0; 53.0] years, and the median duration of the disease was 4.5 [3.0; 10.0] years. The control group for the first stage of the work consisted of 11 healthy donors (median age was 32.0 [24.0; 40.0] years).

When studying the quantitative levels of selected proteins, a correlation with clinical disease characteristics was identified in a number of cases. Therefore, for this stage of the study, several additional clinical groups were included. Specifically, all patients with SCZ, both simple and paranoid, were divided into groups based on their predominant symptomatology and scores on the PANSS scale. Positive symptoms in the general group of patients with SCZ were mild according to the PANSS scale, with an average total score of 18.9 ± 6.2. Negative symptoms were more severe, at 26.4 ± 6.6 points. The average total score for general psychopathological symptoms was 48.2 ± 11.5. The average total PANSS score for SCZ severity was 93.7 ± 20.05 points. Accordingly, negative symptoms predominated in the overall group of patients. Therefore, all schizophrenia patients were divided into groups with predominant (leading) negative (29.5 ± 7.6 points) and positive (24.5 ± 5.3 points) symptoms. Furthermore, some parameters were correlated with whether patients were in the active phase of the disease or in therapeutic remission. Physicians assessed this condition based on the patient’s clinical characteristics.

The study followed the ethical principles of the Declaration of Helsinki. All participants gave written informed consent. Inclusion criteria for patients: age 18–55 years, verified psychiatric diagnosis, and signed informed consent. Inclusion criteria for healthy controls: sex and age matching with patient groups, no history of psychiatric disorders, and signed informed consent. General exclusion criteria for all groups: age <18 or >55 years, comorbid neurological diseases, or additional psychiatric disorders. Individuals with acute or exacerbated chronic infectious, inflammatory, or autoimmune diseases at examination, or those who had experienced acute infections less than four weeks prior, were excluded. Additional exclusion criteria included use of medications or psychoactive substances, sexually transmitted diseases, or refusal to participate.

### 2.2 Collection of Biological Material (Serum)

Blood was collected upon admission before treatment initiation. Venous blood was collected in the morning after an overnight fast using BD Vacutainer Serum Tubes with a clot activator (Becton Dickinson, Franklin Lakes, NJ, USA). Serum was obtained by centrifugation at 2000 ×g for 20 min (Digicen 21R centrifuge, Orto Alresa, Madrid, Spain), then aliquoted and stored at −80 °C until analysis. Due to the different volumes of serum collected from each participant and the gradual depletion of samples during subsequent analyses, the number of participants in the analysed subgroups varied.

### 2.3 Sample Preparation for Mass Spectrometry

#### 2.3.1 Affinity Chromatography for the Removal of Major Proteins

Serum samples were diluted 5-fold with phosphate-buffered saline (PBS), centrifuged at 16,000 rpm for 1 min at 4 °C (Zentrifuge Z 36 HK centrifuge, Hermle Labortechnik GmbH, Wehingen, Germany), and filtered through a 22‑µm syringe filter (Filtropur S, Sarstedt, Nümbrecht, Germany). The supernatant was processed on an ÄKTA Pure chromatograph (GE Healthcare, Chicago, IL, USA) using affinity columns to deplete major proteins: a Multiple Affinity Removal Column Human 14 (Agilent, Santa Clara, CA, USA) for SCZ samples, and a Multiple Affinity Removal HSA/IgG column (4.6 × 50 mm, Agilent, CA, USA) for albumin and IgG for BD samples. Diluted samples (350 µL) were loaded with a PBS buffer (pH 7.4) at 1 mL/min (2–5 min), and the flow-through was collected. The samples were concentrated via ultrafiltration with 3 kDa Microcon® filters (Millipore, Molsheim, France) at 14,000 rcf for 15 min at 20 °C. Protein concentration was determined by 280/260 nm absorbance using a Varioskan LUX spectrophotometer (Thermo Fisher Scientific, Waltham, MA, USA) at the core facility of Medical Genomics (TNRMC).

#### 2.3.2 One-Dimensional Laemmli Gel Electrophoresis

Samples were mixed with bromophenol blue loading buffer, boiled at 95 °C for 5 min, and loaded onto a 12% SDS-polyacrylamide gel (Laemmli method) at 50–100 μg total protein per lane. PageRuler unstained protein standards (10–200 kDa, Thermo Fisher Scientific, Waltham, MA, USA) were used as markers. Electrophoresis was performed in a Protean II xi Cell (Bio-Rad) at 150–180 V using a PowerPac™ Universal Power Supply (Bio-Rad, Hercules, CA, USA). Gels were stained with Coomassie Brilliant Blue (G250) and imaged with an iBright FL1500 Imaging System (Thermo Fisher Scientific, Waltham, MA, USA) at the Medical Genomics core facility (TNRMC). Molecular weights were determined using iBA Analysis Software (Thermo Fisher Scientific, Waltham, MA, USA), and target bands were excised with a scarifier. Eight to twenty samples from different bands per patient were included. The Coomassie dye was removed by washing three times with 50 mM ammonium bicarbonate in 50% acetonitrile on a shaker for 10–15 min until destaining was complete.

#### 2.3.3 Enzymatic Hydrolysis and Extraction of Peptides

Protein digestion was performed using sequencing-grade modified trypsin (#V511A, Promega, Madison, WI, USA), diluted with the supplied buffer (50 mM CH_3_COOH) to 0.01–0.025 µg/mL. 20 μl of trypsin solution was added to the samples and incubated at 4 °C for 1 hour to swell the gel, and then for 18 hours at 37 °C for complete hydrolysis. After stopping the reaction with 25 mM NH_4_HCO_3_, the peptide mixtures were extracted from the gels three times with 50% acetonitrile in 5% formic acid. The resulting extracts were lyophilised and frozen until analysis.

### 2.4 Panoramic Mass Spectrometry

Panoramic mass spectrometry analysis was used as a qualitative method during the initial screening stage to detect differentially expressed proteins. Comparative proteomic analysis of blood serum from SCZ patients and healthy individuals was performed at the “Human Proteome” Core Facility, Institute of Biomedical Chemistry, Moscow. Mass spectrometry (MS) was conducted using an UltiMate 3000 system coupled to an Orbitrap Q‑Exactive HF mass spectrometer (Thermo Fisher Scientific, Rockwell, IL, USA). Peptides were separated on an Agilent Zorbax 300SB‑C18 column (C18 3.5 µm, 75 µm × 150 mm, Agilent Technologies, Santa Clara, CA, USA) with a 65‑min linear gradient. MS data was acquired in positive ion and data-dependent acquisition modes with an Orbitrap high-resolution of 30,000 (MS) and 17,500 (MS/MS).

Proteomic analysis of blood serum from BD patients and healthy individuals was performed at the Advanced Mass Spectrometry Core Facility, Skolkovo Institute of Science and Technology (Moscow), following a protocol presented in [38] with minimal modifications. Tryptic peptides were analysed using an Ultimate 3000 RSLC nano HPLC system coupled to a TIMS‑TOF PRO mass spectrometer (Bruker Corporation, Billerica, MA, USA), using a C18 column (75 µm × 250 mm, 1.7 µm particle size, IonOpticks Aurora Ultimate Column, ESI Source Solutions, Melbourne, Australia). Samples were diluted in 30 µL of 0.1% formic acid, and one microlitre of peptides was analysed using a 90‑min linear gradient of ACN in water with 0.1% formic acid. Mass-spectrometric measurements were carried out using the PASEF acquisition method and data-dependent acquisition mode. The total cycle time was set to 1.16 s, and the number of PASEF MS/MS scans was set to 10. ESI source settings were as follows: 1500 V capillary voltage, 500 V end plate offset, and 3.0 L/min of dry gas at a temperature of 180 °C. The measurements were carried out over the m/z range from 100 to 1700. The range of ion mobilities included values from 0.60 to 1.60 V s/cm^2^(1/k0).

### 2.5 Protein Identification

Protein identification was performed using MASCOT software (version 2.1, Matrix Science, London, UK) against the UniProtKB database with the Homo sapiens taxonomic filter applied. Search parameters included trypsin as the cleavage enzyme, a monoisotopic peptide mass tolerance of ±10 ppm, an MS/MS tolerance of ±0.01 Da, and one missed cleavage allowed. Variable modifications were cysteine propionamide and methionine oxidation. Positive identification criteria were set as follows: peptide and protein FDR <0.01, at least two matched peptides per protein, and a minimum protein score >30. To account for the use of different mass spectrometry platforms and to eliminate potential batch effects, an intra-sample rank-based normalisation (non-parametric percentile scaling) was applied to the protein score matrix prior to downstream statistical analysis. Absolute MASCOT scores within each individual serum run were transformed into relative fractional ranks ranging from 0 (lowest score) to 1 (highest score) and scaled by the total number of identified proteins in that specific sample. This non-parametric normalisation utilised the stable internal hierarchy of the major serum proteins (e.g., albumin, immunoglobulins) as baseline anchors, ensuring full cross-platform compatibility of the datasets.

### 2.6 Determination of NMDAR1 Concentration in Serum by the Targeted Mass Spectrometry

The targeted mass spectrometry method with selected reaction monitoring (SRM) was applied to analyse the NMDAR1 content in the serum using a synthetic isotope-labelled standard NMDAR1 peptide (SIS). The SIS peptide IVNIGAVLSTR at a concentration of 50 fmol/µL and the SIS peptide IPVLGLTTR at a concentration of 125 fmol/µL were used. Chromatographic separation was performed using an UltiMate 3000 system coupled to a triple quadrupole mass spectrometer TSQ Vantage (Thermo Fisher Scientific, Rockwell, IL, USA). Peptides were separated on a ZORBAX SB‑C18 analytical column (150 × 0.5 mm, 5 µm particle size, Agilent Technologies, Santa Clara, CA, USA) using an acetonitrile gradient at a flow rate of 20 µL/min. Mass spectrometric analysis was conducted in dynamic selected reaction monitoring (dSRM) with the isolation window for the first and third quadrupoles being 0.7 Da, and the scan cycle time was 2 s. Quantification of target peptide concentrations was performed automatically using Skyline MacCoss Lab Software (version 4.1.0, MacCoss Lab., Seattle, WA, USA), followed by manual verification of the results.

### 2.7 Determination of the Concentration of Target Proteins by ELISA

Quantitative determination of the studied protein levels in the serum was performed using an enzyme-linked immunosorbent assay (ELISA). The following commercial kits were used to determine the concentration of five proteins:

(1) ELISA Kit for Dermcidin (DCD) (SEC896Hu, Cloud-Clone Corp., Katy, TX, USA), Limit of Detection (LOD): 0.017 ng/mL, Lower Limit of Quantification (LLOQ): 0.05 ng/mL, detection range: 0.05–50 ng/mL;

(2) ELISA Kit for Low Density Lipoprotein Receptor Related Protein 2 (LRP2) (SED101Hu, Cloud-Clone Corp., Katy, TX, USA), LOD: 13.8 pg/mL, LLOQ: 31.2 pg/mL, detection range: 31.2–2000 pg/mL;

(3) ELISA Kit for Ankyrin Repeat Domain Protein 12 (ANKRD12) (SEM789Hu, Cloud-Clone Corp., Katy, TX, USA), LOD: 0.118 ng/mL, LLOQ: 0.312 ng/mL, detection range: 0.312–20 ng/mL;

(4) Human Ankyrin 3 (ANK3) ELISA Kit (E01A1450, Shanghai BlueGene Biotech CO., LTD., Zhoupu Town, Pudong New Area, Shanghai, China), LOD: 0.1 ng/mL, LLOQ: 0.5 ng/mL, detection range: 0.5–10 ng/mL;

(5) ELISA kit for N-methyl-D-aspartate receptor (NMDAR) subunit 1 (NMDAR1) (GRIN1) (SEE805HU, Cloud-Clone Corp., Katy, TX, USA), LOD: 0.118 ng/mL, LLOQ: 0.312 ng/mL, detection range: 0.312–20 ng/mL.

In addition, the concentration of antibodies to NMDAR was studied using the anti-NR2 antibody assay ELISA kit (RDS-LAB LLC, Moscow, Russia) (according to the manufacturer, the target antigens are NMDAR2A/2B).

The marker analysis was carried out in accordance with the kit manufacturer’s recommendations using a Varioskan LUX spectrophotometer (Thermo Scientific, Waltham, MA, USA) located at the Medical Genomics core facility (TNRMC). The values obtained below the LOD were considered equal to zero.

### 2.8 Statistical Analysis

Mass spectrometric analysis results were analysed in R in RStudio (Version: 2026.01.2+418) (http://www.r-project.org). Differentially expressed proteins were assessed by comparing identified proteins in the analysed groups using Fisher’s exact test with Yates’s correction. Differences in the quantitative analysis of selected proteins across several independent groups were assessed using the nonparametric Kruskal-Wallis test with Mann-Whitney post-hoc test and the Bonferroni correction for multiple comparisons. The Bonferroni correction was applied when comparing each protein separately (three pairwise comparisons, *p* < 0.0167 were considered significant). Categorical variables (detection frequency) were assessed using the chi-square test or Fisher’s exact test with Yates’ correction. Statistical data processing was performed using STATISTICA 10.0 (StatSoft, Tulsa, OK, USA). OriginPro (Version: 2021, OriginLab Corporation, Northampton, MA, USA) was used for plotting.

## 3. Results

### 3.1 Differentially Expressed Proteins of the Study Groups Identified Based on Mass Spectrometric Analysis

In the first stage, mass spectrometric analysis was used as a qualitative method to assess the frequency of protein detection between groups and to generate a list of potential biomarkers. A comparative proteomic analysis of serum proteins from SCZ patients and healthy individuals using panoramic mass spectrometry yielded a total of 4555 proteins. Of these, 4334 proteins were identified in the control group and 4115 proteins were identified in the general group of SCZ patients. Of these proteins, 440 were unique to the control group and 221 were unique to SCZ patients (Fig. 1A).

**Fig. 1.**
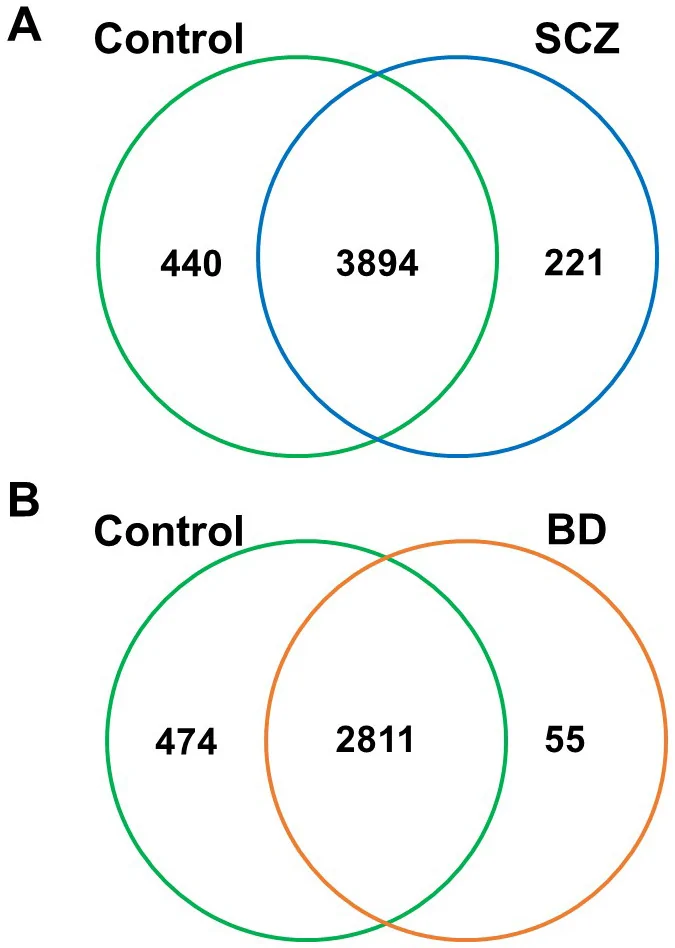
**Venn diagram showing the overlap of proteins identified by proteomic analysis**. (A) Comparison of identified proteins in the SCZ and healthy control groups. (B) Comparison of identified proteins in the BD and healthy control groups. SCZ, schizophrenia; BD, bipolar disorder.

A comparative proteomic analysis of serum proteins from BD patients and healthy individuals identified a total of 3340 proteins. 3285 proteins were identified in healthy individuals and 2866 in patients with BD. Of these, 55 unique proteins were identified in BD patients, while 474 proteins not found in BD patients were identified in healthy individuals (Fig. 1B).

A summary table of proteins identified by panoramic mass spectrometry can be found in the Figshare data repository (https://doi.org/10.6084/m9.figshare.32796687).

Based on these results, five proteins showing differential expression and consistently high normalized Mascot scores were selected from the entire dataset for further quantitative analysis. The selection was primarily based on literature or first-hand data on the known or suspected association of the protein with SCZ or BD, as well as its functional activity and potential involvement in the pathogenesis of mental disorders (Table 1, Ref. [24,25,26,29,30,35,36,39,40,41,42,43,44,45,46,47,48]). Table 2 presents the characteristics of the proteins selected for quantitative analysis based on the obtained mass spectrometry results. In the second stage, the selected proteins were analyzed using quantitative methods.

**Table 1.** **Differentially expressed proteins selected for quantitative analysis: literature data**.

Protein/alternative name	Uniprot ID	Gene name	Function and expression pattern	References
NMDAR1/GluN1 NMDA1	Q05586	*GRIN1*	NMDAR subunit is involved in synaptic plasticity and is expressed primarily in neurons of the cerebral cortex and hippocampus.	[25,26]
Ankyrin 3/ankyrin-G	Q12955	*ANK3*	A cytoskeletal protein that organises axon structure and nodes of Ranvier, regulates GABA binding to its receptors, and stabilises their structure. Several isoforms are expressed in the brain.	[35,36,39,40]
ANKRD12	Q6UB98	*ANKRD12*	A protein with ankyrin repeat motifs that inhibits the transcriptional activity of nuclear receptors through interaction with histone deacetylases; it is expressed in the brain, particularly in the cerebellum.	[24,41,42]
Dermcidin	P81605	*DCD*	A protein with bactericidal activity, but also active in OS and inflammation. Diffusible survival-promoting peptide (Y-P30), derived from DCD, promotes neuronal survival during injury. It is expressed in the pons and paracentral gyrus of the brain.	[29,43,44,45]
LRP2/Megalin	P98164	*LRP2*	An endocytic receptor involved in the transport of various ligands across the BBB and BCB; it is expressed in the choroid plexus epithelium, ependymal cells of the subventricular zone, and various neuronal subpopulations, where it regulates stem cell proliferation.	[30,46,47,48]

Abbreviations: NMDAR, N-methyl-D-aspartate receptor; *LRP2*, low-density lipoprotein receptor-related protein 2; *DCD*, dermcidin; *ANKRD12*, ankyrin repeat domain-containing protein 12; GABA, gamma-aminobutyric acid; BBB, blood-brain barrier; BCB, blood-cerebrospinal fluid barrier.

**Table 2.** **Differentially expressed proteins selected for quantitative analysis: proteomic analysis**.

UniProt ID	Protein name	Normalized SCZ MASCOT score	Normalized BD MASCOT score	Normalized control MASCOT score	Delta rank	
					SCZ - Control	BD - Control
P81605	Dermcidin	0.8257	0.8929	0.9678	–0.1422	–0.0753
P98164	Low-density lipoprotein receptor-related protein 2	0.6150	0.8590	0.6073	0.0077	0.2518
Q05586	Glutamate receptor ionotropic, NMDA 1	0.4243	0.3639	-	-	-
Q12955	Ankyrin-3	0.4951	0.1781	0.8009	–0.3058	–0.6229
Q6UB98	Ankyrin repeat domain-containing protein 12	0.9991	0.9158	0.8917	0.1074	0.0239

### 3.2 Quantitative Assessment of a Number of Selected Proteins in Serum

#### 3.2.1 N-methyl-D-aspartate Receptor 1 (NMDAR1)

Serum NMDAR1 protein concentration was measured by ELISA (Fig. 2A). However, no significant differences were found between the analysed groups (Kruskal-Wallis ANOVA, *p* = 0.98). NMDAR1 protein was undetectable in approximately 50% of participants from all groups. Differences were assessed taking into account positive and negative NMDAR1 protein concentration values. The groups of patients and healthy controls did not differ significantly in the frequency of NMDAR1-positive and -negative participants (Fig. 2B). The NMDAR1 level in SCZ patients with dominant positive symptoms did not differ from that in patients with negative symptoms (*p* = 0.97). Furthermore, the NMDAR1 levels were significantly elevated in BD patients with a current depressive episode compared to patients with a current mixed episode (Fig. 2C), but after the Bonferroni correction, the differences lost significance. Therefore, these results can only be considered as a trend.

**Fig. 2.**
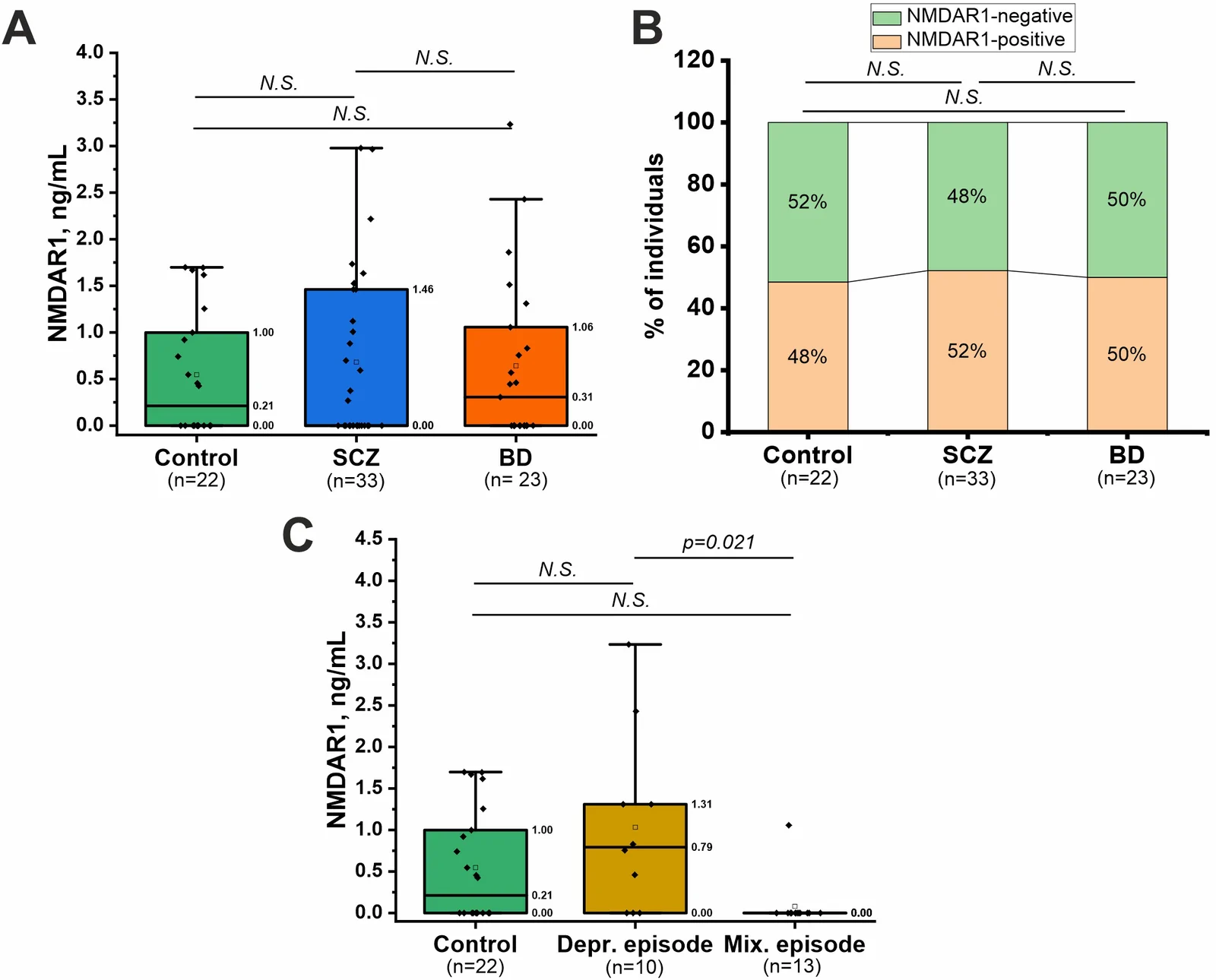
**Serum NMDAR1 concentrations in SCZ and BD patients and healthy controls measured by ELISA**. (A) NMDAR1 concentrations in the analysed groups. (B) Frequency of NMDAR1-positive and NMDAR1-negative samples in SCZ, BD, and healthy controls. (C) NMDAR1 levels in the control group and in BD patients with current depressive and mixed episodes. The significance of differences was calculated using the Kruskal-Wallis test with the Mann-Whitney post-hoc test (A,C) and the chi-square test with Yates’ correction (B). Numerical values to the right of the boxplots represent the median, Q1, and Q3. ELISA, enzyme-linked immunosorbent assay; N.S., not significant.

Since NMDAR1 levels were below the detection limit in approximately half of the cases, it was assumed that the ELISA sensitivity was insufficient. Therefore, a highly sensitive quantitative proteomics method was applied to measure NMDAR1 protein concentration. Serum NMDAR1 protein levels were measured using the SRM method, using synthetic standard NMDAR1 peptides.

Fig. 3 shows the results of the SRM analysis. For the NMDAR1 protein (Q05586), a signal from the native peptide IVNIGAVLSTR (Fig. 3A) and a signal from the standard SIS peptide (Fig. 3B) were detected in the matrix.

**Fig. 3.**
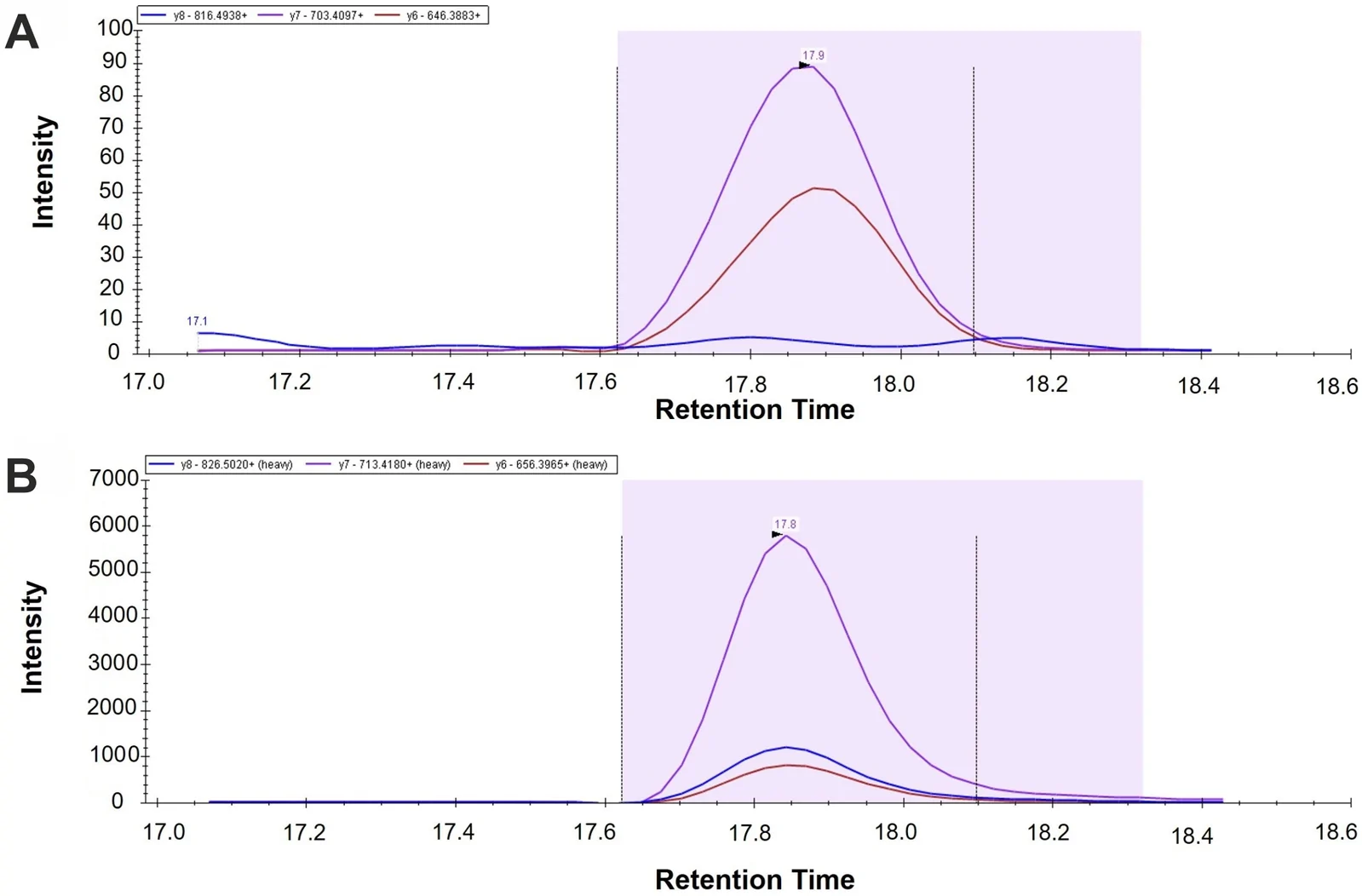
**Visualisation of the results of SRM analysis of synthetic and native precursors for the peptide IVNIGAVLSTR (Q05586 NMDAR1)**. Extracted ion current chromatograms of fragment ions in the matrix: (A) native precursor, (B) synthetic standard precursor (x-axis – retention time, y-axis – signal intensity). The purple line is the quantifier (y7, m/z, 703.4097); blue (y8, m/z, 816.4938) and red (y6, m/z, 646.3883) are qualifiers. Data was visualised in Skyline MacCoss Lab Software (version 4.1.0). SRM, selected reaction monitoring.

SRM analysis revealed significant differences in NMDAR1 protein levels between the analysed groups (Kruskal-Wallis ANOVA, *p* = 0.024). Specifically, NMDAR1 levels were significantly increased in BD compared to healthy controls (*p* = 0.021) (Fig. 4A), but after the Bonferroni correction, the differences were no longer significant, indicating only a trend. However, the frequency of NMDAR1-positive samples from BD patients was also significantly higher than in healthy controls (Chi-square with Yates’ correction, *p* < 0.05) (Fig. 4B).

**Fig. 4.**
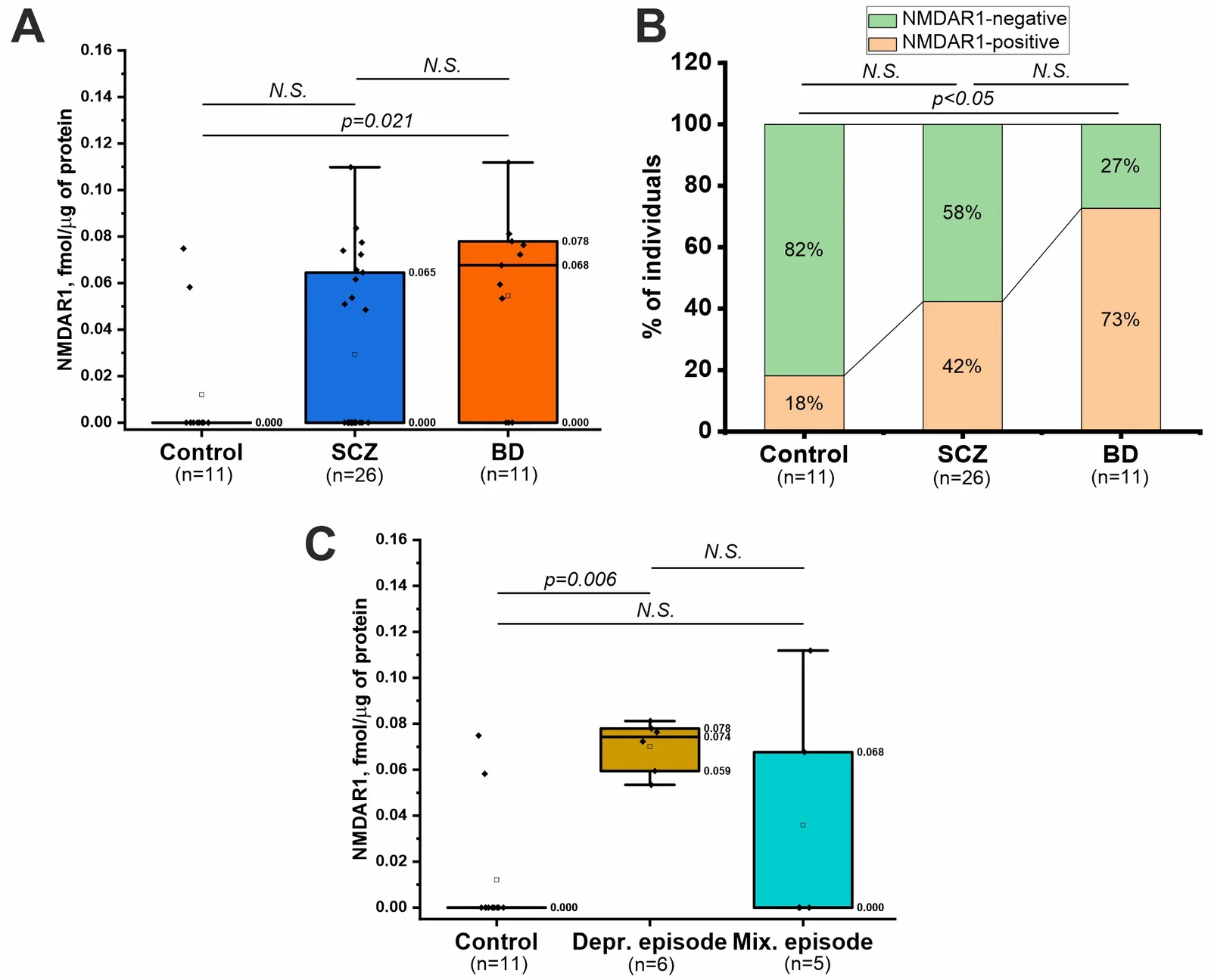
**Serum NMDAR1 levels in SCZ and BD patients and healthy controls assessed by SRM**. (A) Serum NMDAR1 levels in the analysed groups. (B) Frequency of NMDAR1-positive and NMDAR1-negative samples in SCZ, BD, and healthy controls. (C) NMDAR1 content in controls and BD patients with current depressive and mixed episodes. The significance of differences was calculated using the Kruskal-Wallis test with Mann-Whitney post-hoc test (A,C) and Chi-square with Yates’ correction (B). Numerical values to the right of the boxplots represent the median, Q1, and Q3. N.S., not significant.

Clinical association analysis revealed no changes in NMDAR1 levels in SCZ depending on the prevailing symptomatology (*p* = 0.68). In the case of BD, although the median NMDAR1 levels in patients with a depressive episode were higher than in patients with a mixed episode (0.07 and 0 fmol/µg, respectively) (Fig. 4C), the differences did not reach statistical significance due to the wide range of values. However, the NMDAR1 level in BD patients with a current depressive episode was significantly higher compared with the controls (Fig. 4C).

Thus, the detection rate of the NMDAR1 protein using the SRM method was higher, at least in the BD patients, which made it possible to obtain a significant increase in its quantity in BD compared to the control group due to the group of patients with a current depressive episode.

#### 3.2.2 Anti-NMDAR Antibodies

Since NMDAR fragments circulating in serum can lead to the generation of anti-NMDAR antibodies, contributing to the development of NMDAR-antibody encephalitis, accompanied by psychotic symptoms [49], the level of anti-NMDAR antibodies was also assessed in this study.

The level of anti-NMDAR antibodies was shown to differ between the groups (Kruskal-Wallis ANOVA, *p* = 0.04) and was significantly higher in patients with SCZ and BD compared to healthy individuals (Fig. 5A), but after the Bonferroni correction, the differences lost significance, so these results should be considered as a trend. Also, the level of antibodies in SCZ and BD did not differ significantly. Next, the mean+3SD value in the healthy sample (>49.7 ng/mL) was chosen as the cutoff point to identify participants with the highest levels of anti-NMDAR antibodies. There were no participants with high levels of anti-NMDAR antibodies among the healthy individuals. The frequency of participants with high levels of antibodies in SCZ was 13%, but no significant differences were found compared to the controls. In BD, 18% of patients had high levels of anti-NMDAR antibodies, and the differences were significant compared to controls (Fig. 5B). Thus, BD patients were more likely to have high levels of anti-NMDAR antibodies.

**Fig. 5.**
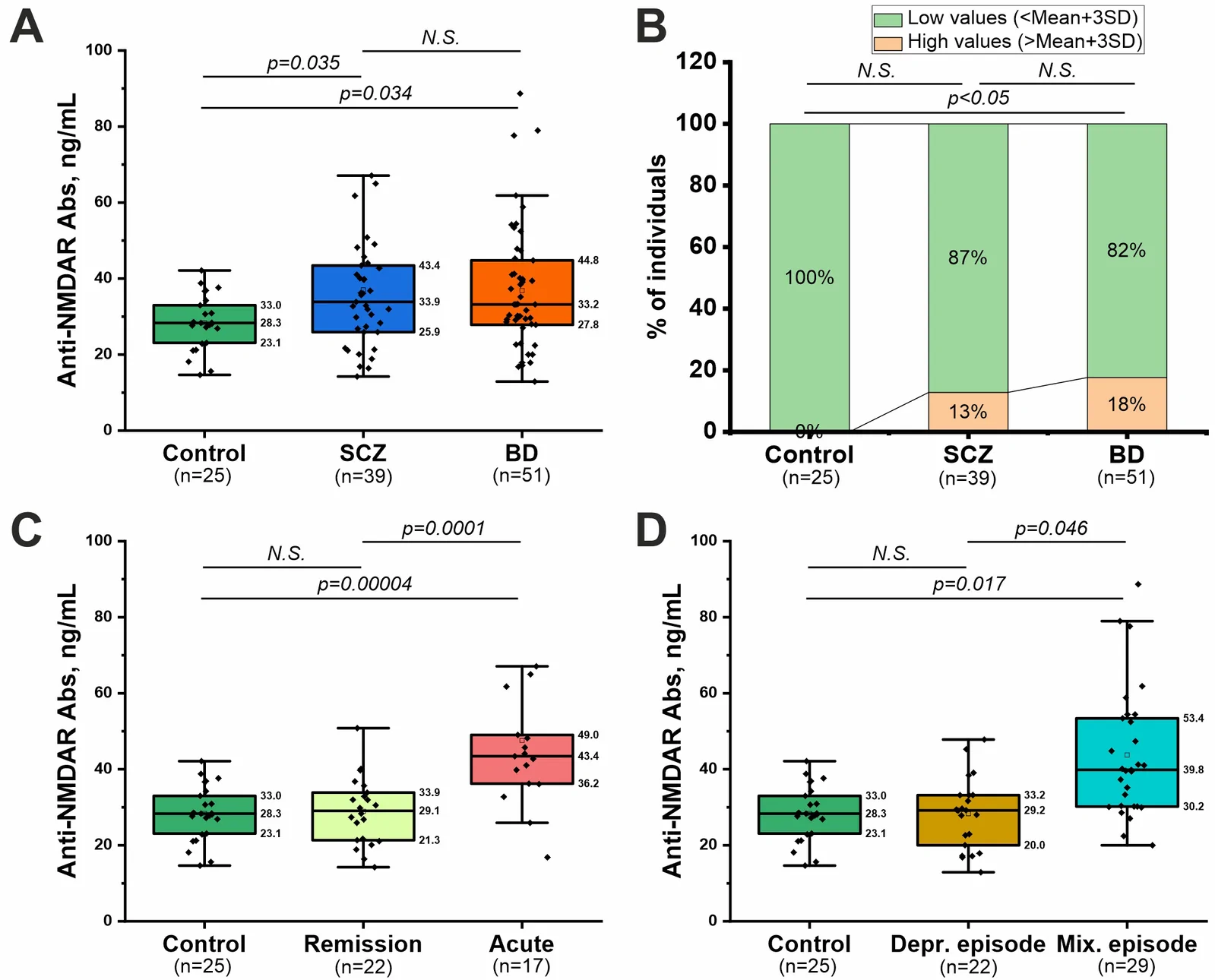
**Concentration of anti-NMDAR antibodies in the serum of SCZ and BD patients and healthy individuals measured by ELISA**. (A) Level of anti-NMDAR antibodies in the analysed groups. (B) Frequency of samples with low (<mean+3SD = 49.7 ng/mL) and high (>49.7 ng/mL) levels of anti-NMDAR antibodies in SCZ, BD, and healthy individuals. (C) Level of anti-NMDAR antibodies in the control group and in SCZ patients in remission and the acute phase of the disease. (D) Level of anti-NMDAR antibodies in the control group and in BD patients with current depressive and mixed episodes. The significance of differences was calculated using the Kruskal-Wallis test with the Mann-Whitney post-hoc test (A,C,D) and Chi-square with Yates’ correction (B). N.S., not significant.

Analysis of clinical associations revealed that SCZ patients in the acute phase of the disease had significantly higher anti-NMDAR antibody levels than patients in remission and the control group (Fig. 5C). Antibody levels in remission did not differ from those in the control group. Moreover, among patients in the acute phase of the disease, 17.6% had high levels (>49.7 ng/mL) of anti-NMDAR antibodies, while only one such patient (4.5%) was found among the patients in remission. There were no significant differences in antibody levels depending on the predominant SCZ symptomatology (*p* = 0.74). Anti-NMDAR antibody levels negatively correlated with the age of SCZ patients (r = –0.34, *p* = 0.03).

In BD, an increase in anti-NMDAR antibodies was found in the BD patients with a current mixed episode compared with a depressive episode and the control group (Fig. 5D), but the Bonferroni correction levelled out these differences. Interestingly, among patients with a mixed episode, 28.6% had high levels (>49.7 ng/mL) of anti-NMDAR antibodies, while there were no such patients in the depressive episode. No other clinical associations were identified.

#### 3.2.3 Ankyrin 3 (ANK3)

ANK3 protein was detected in all samples. ANK3 concentrations differed significantly between the study groups (Kruskal-Wallis ANOVA, *p* = 0.028) and were significantly lower in both SCZ (*p* = 0.01) and BD (*p* = 0.03) compared to the controls (Fig. 6A), but after the Bonferroni correction, the decrease in BD patients lost significance, although the decrease in SCZ remained significant. ANK3 levels did not differ between the SCZ and BD groups. Thus, ANK3 levels decreased in SCZ and at the trend level in BD compared to controls.

**Fig. 6.**
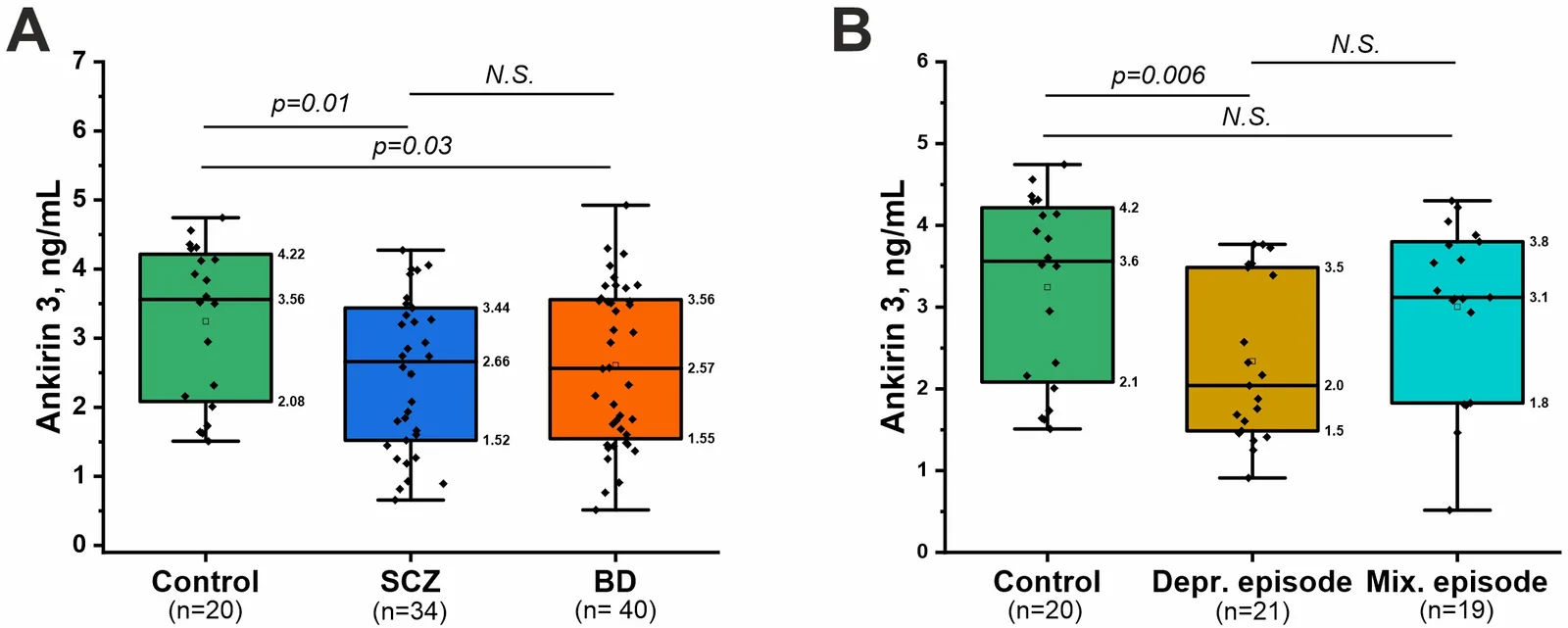
**Serum ANK3 concentrations in SCZ and BD patients and healthy individuals measured by ELISA**. (A) ANK3 concentrations in the analysed groups. (B) ANK3 levels in the control group and in BD patients with current depressive and mixed episodes. The significance of differences was calculated using the Kruskal-Wallis test with the Mann-Whitney post-hoc test. ANK3, Ankyrin 3; N.S., not significant.

Clinical association analysis revealed no differences in ANK3 levels between BD patients with a current depressive episode and a mixed episode (Fig. 6B), but ANK3 levels in BD patients with a current depressive episode were significantly lower than in the controls. Also, no associations with the leading symptoms of SCZ were found (*p* = 0.45).

#### 3.2.4 Ankyrin Repeat Domain-Containing Protein 12 (ANKRD12)

ANKRD12 concentrations differed significantly between groups (Kruskal-Wallis ANOVA, *p* = 0.017). The highest median ANKRD12 concentration was detected in the serum of SCZ patients (Fig. 7A). The ANKRD12 levels in SCZ were significantly higher than in the control group, while in BD, they were no different from the control. In some samples, ANKRD12 levels were below the detection limit, but the frequency of ANKRD12-positive and -negative samples did not differ between the groups (Fig. 7B).

**Fig. 7.**
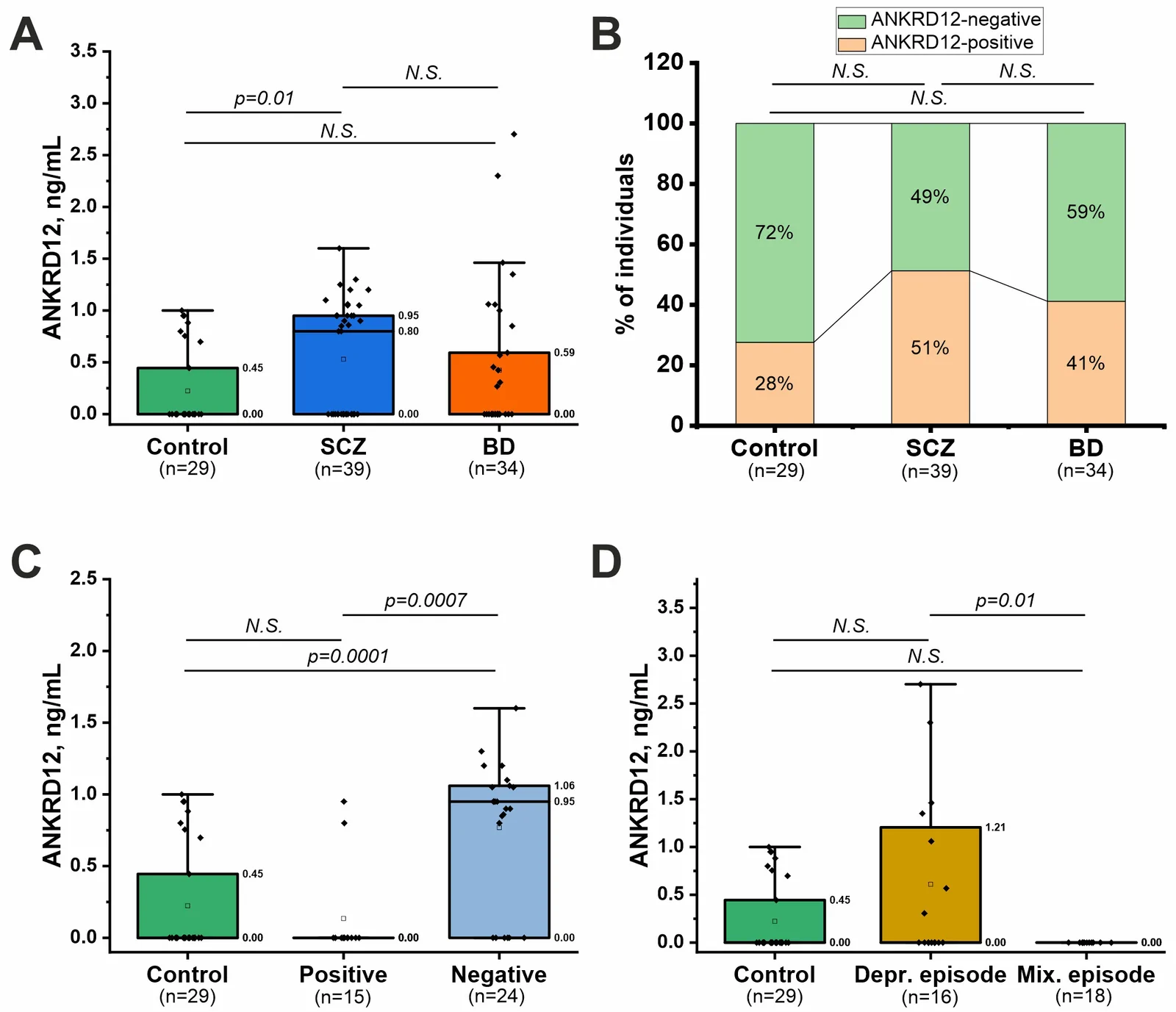
**ANKRD12 concentration in the serum of SCZ and BD patients and healthy individuals by ELISA**. (A) ANKRD12 level in the analysed groups. (B) Frequency of ANKRD12-positive and ANKRD12-negative serum samples in the group of SCZ and BD patients and healthy individuals (control). (C) ANKRD12 concentration in the serum of SCZ patients with leading positive and negative symptoms. (D) ANKRD12 level in the control group and in BD patients with current depressive and mixed episodes. The significance of differences was calculated using the Kruskal-Wallis test with the Mann-Whitney post-hoc test (A,C,D) and the Chi-square with Yates’ correction (B). N.S., not significant.

Clinical association analysis revealed a significant decrease in ANKRD12 in SCZ patients with dominant positive symptoms (Fig. 7C) and an increase in patients with dominant negative symptoms, including those compared with controls. Furthermore, ANKRD12 levels were higher in BD patients with a current depressive episode compared with those with a mixed episode (Fig. 7D). Thus, ANKRD12 concentrations increased to a greater extent in SCZ patients, and this increase was associated with dominant negative symptoms.

#### 3.2.5 Dermcidin (DCD)

The DCD concentration did not differ significantly between the study groups (Kruskal-Wallis ANOVA, *p* = 0.19) (Fig. 8A), and about half of the samples had undetectable DCD levels. The frequency of DCD-positive and DCD-negative samples also did not differ significantly between the groups (Fig. 8B). The DCD level in SCZ patients in remission was significantly lower than in patients in the acute phase of the disease and the control group (Fig. 8C). In BD patients, the frequency of DCD-positive samples in BD with a current depressive episode was significantly higher than in patients with a mixed episode (*p* = 0.008) (Fig. 8D). No other significant differences were found.

**Fig. 8.**
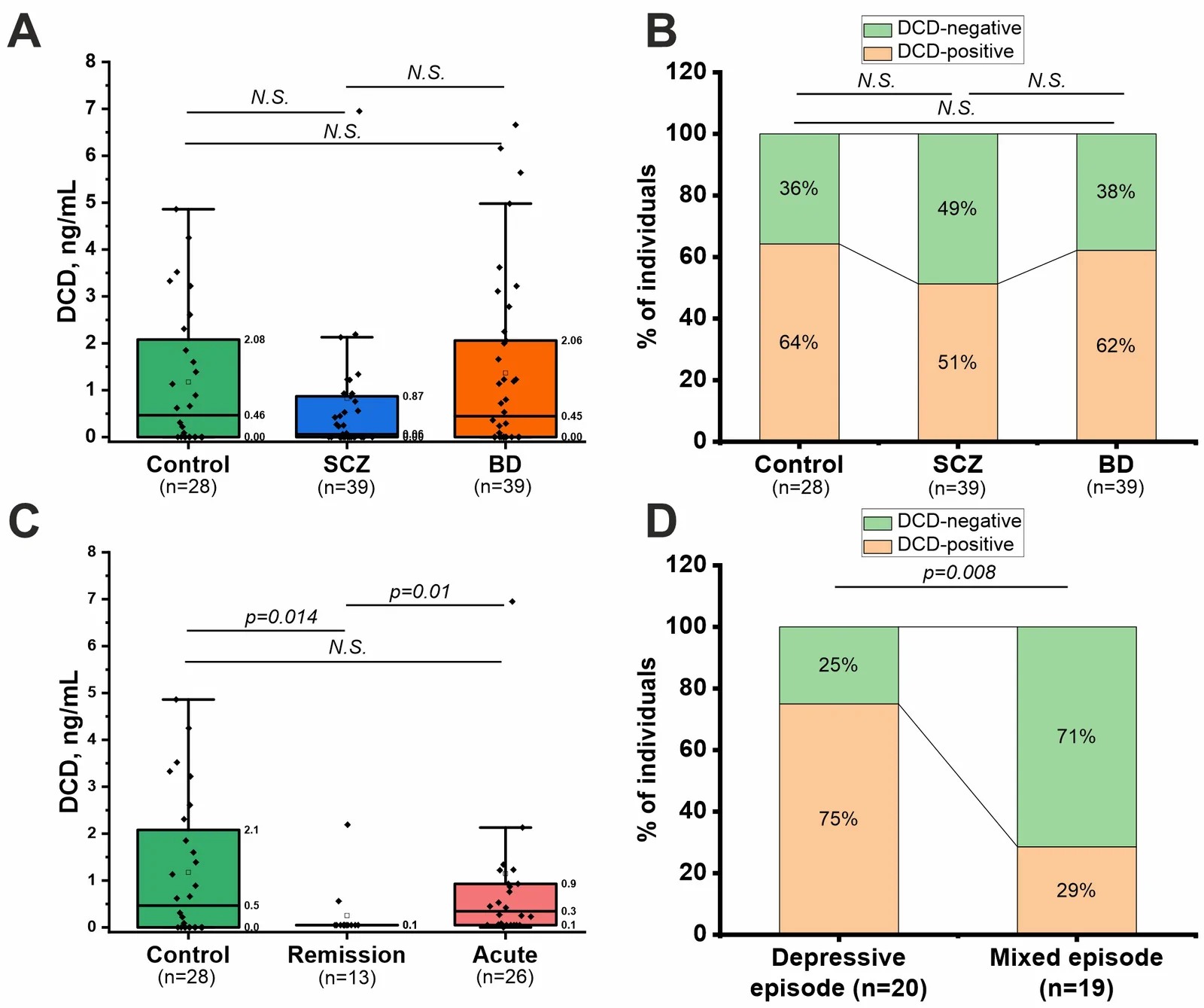
**Serum dermcidin (DCD) concentrations in SCZ and BD patients and healthy controls measured by ELISA**. (A) DCD levels in the analysed groups. (B) Frequency of dermcidin (DCD)-positive and DCD-negative serum samples in SCZ and BD patients and healthy controls measured by ELISA. (C) DCD levels in the control group and in SCZ patients in remission and the acute disease phase. (D) Frequency of DCD-positive and DCD-negative samples in BD patients with current depressive and mixed episodes. The significance of differences was calculated using the Kruskal-Wallis with the Mann-Whitney post-hoc test (A,C) and the Chi-square test with Yates’ correction (B,D). N.S., not significant.

#### 3.2.6 Low-Density Lipoprotein Receptor-Related Protein 2 (LRP2)

LRP2 protein was detected in all samples tested. LRP2 concentrations differed significantly between all groups (Kruskal-Wallis ANOVA, *p* = 0.01). LRP2 levels were increased approximately 2-fold in BD patients and approximately 1.5-fold in SCZ patients compared to healthy controls (Fig. 9A).

**Fig. 9.**
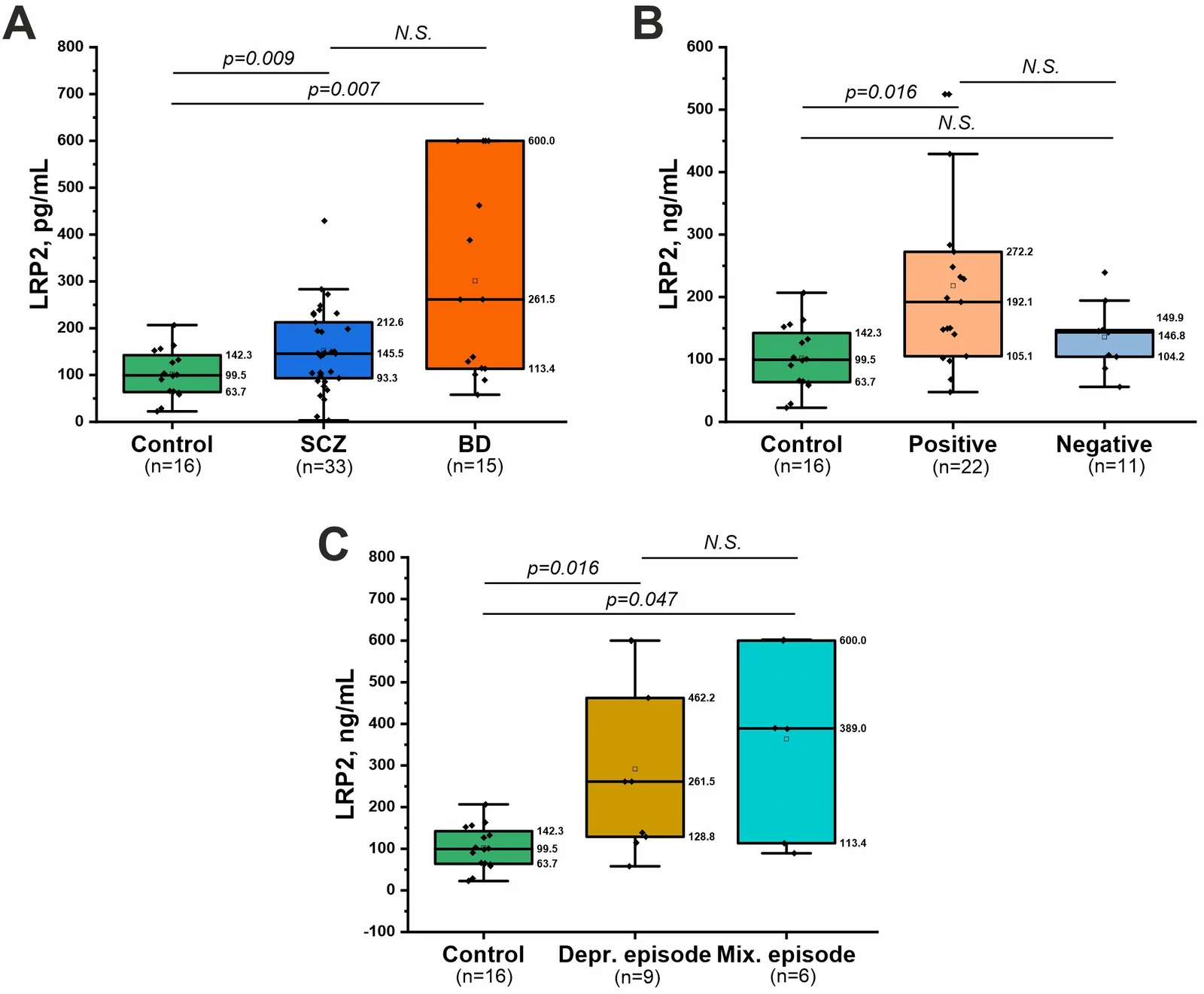
**LRP2 concentration in the serum of SCZ and BD patients and healthy individuals measured by ELISA**. (A) LRP2 concentration in the analysed groups. (B) LRP2 level in the control group and SCZ patients with predominant positive and negative symptoms. (C) LRP2 level in the control group and in BD patients with current depressive and mixed episodes. The significance of differences was calculated using the Kruskal-Wallis test with the Mann-Whitney post-hoc test. N.S., not significant.

A subgroup analysis of SCZ patients revealed the highest LRP2 levels in patients with predominantly positive symptoms compared to controls (Fig. 9B). Differences in LRP2 levels between patients with predominantly positive and negative symptoms did not reach statistical significance (*p* = 0.11).

In BD, LRP2 levels were higher in patients with a current depressive and mixed episode compared to controls (Fig. 9C), but after the Bonferroni correction, the increase in BD patients with a mixed episode lost significance. LRP2 levels did not differ between different BD episodes. Thus, an increase in LRP2 was observed in BD patients with current depression and at the trend level in a mixed episode compared to controls.

## 4. Discussion

In this study, five minor proteins involved in neuronal functions were selected from a list of differentially expressed serum proteins in SCZ and BD patients for further quantitative analysis. Most of these proteins have been previously associated with mental disorders, but their pathogenetic roles and quantitative changes remain unclear [27,29,30,31,32,33]. Although their serum concentrations were low, we identified significant changes in certain patient subgroups.

Analysis of NMDAR1 fragments in blood serum was considered the most promising. Combining ELISA and MS results, we can conclude that NMDAR1 levels are significantly elevated in BD patients with a current depressive episode (Figs. 2,4). Glutamate receptor fragments are thought to enter the circulation due to synaptic degradation or neuronal damage [16,50], therefore, elevated NMDAR1 fragments may reflect synaptic degradation and BBB dysfunction in BD patients. However, this hypothesis requires experimental verification. In addition, no significant increase was detected in SCZ patients, and in approximately half of the samples, levels were below the detection limit in ELISA and SRM analysis. These results may be explained by insufficient ELISA sensitivity, differential epitope recognition, protein degradation, and polymorphic variants of NMDAR subunit genes affecting the amino acid sequence and structure of NMDAR1, thereby complicating detection [51,52]. But SRM analysis is free from most of these limitations. Thus, NMDAR1 may appear in the blood only in a subset of patients.

We observed a significant decrease in serum ANK3 levels in BD patients with a current depressive episode and in SCZ patients (Fig. 6). ANK3 is a critical structural component of both synapses and axon hillocks, and its deficiency reflects a general reduction in interneuronal contact density [53]. Mutations in the *ANK3* gene are among the most reproducible risk factors for these disorders in genome-wide association studies [54,55]. Disruption of spine architecture due to ANK3 deficiency impairs long-term potentiation, manifesting as reduced neuroplasticity and emotional lability [53,56]. Smith et al. [40] demonstrated that the 190 kDa isoform of ANK3 acts as a perisynaptic barrier, physically restricting lateral receptor diffusion. This interaction is critical for NMDARs: while NMDAR1 (GluN1) ensures basic ion channel formation, NMDAR2 (GluN2A/B) regulatory subunits interact with the cytoskeleton via their C-terminal domains. Disruption of ANK3 expression destabilises the postsynaptic density [40]. ANK3 contributes to synaptic stability by forming specific nanodomains in dendritic spines where NMDAR subunits participate in glutamatergic synapse integrity. In the absence of ANK3 anchoring, receptors shift from synaptic to extrasynaptic membrane regions, exposing regulatory sites on the GluN2B subunit. Normally, this motif is hidden by scaffold proteins, but loss of this interaction initiates receptor internalisation [57]. This process explains the NMDAR hypofunction underlying negative symptoms and cognitive deficits in SCZ [10].

Elevated NMDAR1 and decreased ANK3 levels in the depressive episode of BD may be associated with intense receptor shedding from neuronal membranes due to synaptic destabilisation. Thus, ANK3 deficiency may result in the release of receptor subunits into peripheral circulation, however, this requires further study. Nevertheless, peripheral blood markers of this process are identified here for the first time.

Thus, the synergy between NMDAR and ANK3 forms a dynamic barrier preventing a pathological receptor “washout” from the membrane. Deficiency of either component shifts neurons from plastic stability toward synaptic degradation, linking SCZ and BD within a unified synaptopathy concept. Further study of mechanisms stabilising NMDAR through ANK3 interactions may offer therapeutic targets for mental disorders.

We also found a trend towards increasing anti-NMDAR antibody levels in both SCZ and BD. Among BD patients, those with mixed episodes showed the highest antibody levels, significantly differing from the controls (Fig. 5). This trend aligns with published data: anti-NMDAR antibodies are detected in 3–4% of SCZ patients [58] and 10% of BD patients [59,60]. This result is consistent with the significant increase in NMDAR-positive samples in BD patients compared to controls (Fig. 4), suggesting that NMDAR1 fragments in serum, due to increased BBB permeability, may trigger autoantibody formation. The combination of elevated NMDAR1 and anti-NMDAR antibodies alongside ANK3 deficiency indicates convergent pathological pathways. Subsequent NMDAR antibody formation may act as a secondary aggravating factor, perpetuating synaptic dysfunction via receptor internalisation.

Interestingly, autoantibodies in BD are significantly elevated in patients with a current mixed episode, whereas elevated NMDAR levels are seen in those with a depressive episode. Anti-NMDA receptor encephalitis, characterised by autoantibody production, shares symptoms with mental disorders, including acute psychosis, delusions, hallucinations, and affective instability [61,62], complicating a differential diagnosis with BD and SCZ. Our findings—significantly elevated anti-NMDAR antibodies specifically in mixed episodes of BD—likely explain these characteristic clinical symptoms.

The detection of NMDAR1 only in a patient subgroup (Fig. 4) suggests a cluster of individuals at increased risk of secondary immune reactions and anti-NMDAR antibody generation (Fig. 5). The negative correlation between anti-NMDAR antibody levels and age in SCZ patients may reflect disease stage-specific immune responses. Identifying patient subgroups with high antibody levels is important for understanding disease aetiology. Thus, the clinical similarity between SCZ and BD may be explained by the convergence of structural abnormalities (ANK3 deficiency), receptor dysfunction and autoimmune reactions.

Another ankyrin protein studied was ANKRD12. Unlike other proteins, ANKRD12 showed a specific association with schizophrenia: the highest median concentration was recorded in the SCZ group, consistent with increased ANKRD12-positive samples in SCZ (Fig. 7). This increase is driven by patients with predominantly negative symptoms, suggesting a role for this biomarker in cognitive and emotional deficits. ANKRD12 influences CNS function through epigenetic regulation, acting as a cofactor that inhibits nuclear receptor transcriptional activity by interacting with HDACs [41]. Postmortem brain studies confirm a dysregulation of HDAC1 and HDAC2 expression in SCZ, BD, and depression [33,63,64,65]. Elevated ANKRD12 levels may enhance HDAC activity, which, in experimental models, suppresses transcription of memory- and learning-related genes. Excessive HDAC activity is associated with increased depressive symptoms and a reduced therapy response [66], while HDAC2 knockdown in the CNS improves spatial, associative, and working memory [67]. Thus, ANKRD12 may mediate “epigenetic priming”, limiting neural network plasticity and contributing to persistent cognitive deficits. The link between ANKRD12 and negative symptoms in schizophrenia is supported by genetic studies associating this gene with progressive cognitive decline and altered white matter integrity [68,69,70].

Serum LRP2 levels were significantly elevated in the overall group of patients with SCZ or BD, and particularly in BD patients with a current depressive episode, compared with the control group (Fig. 9). Patients with predominantly positive symptoms also showed significant increases compared to controls. LRP2 is a large multifunctional receptor involved in endocytosis and cellular signalling. Previously linked to SCZ pathogenesis [30], this protein has not been mentioned in the context of BD. LRP2 is expressed in choroid plexus epithelium, brain endothelial cells, astrocytes, spinal cord, and retinal ganglion cells, where it activates neurotrophic factor synthesis [71,72]. Its biological significance may relate to neuroprotection and maintenance of brain structural plasticity [73]. Whether serum soluble LRP2 levels reflect brain changes remains uncertain, but our results highlight this protein as an important pathogenetic and possibly etiological factor in mental disorders.

We found no significant differences in serum DCD levels between study groups, except for a high detection rate in the depressive episode of BD (Fig. 8). DCD, an antimicrobial peptide [29], is a stress-induced protein that stimulates tumour necrosis factor-alpha synthesis, a key proinflammatory cytokine [74,75]. The absence of significant DCD differences and a low detection rate suggest that neuroinflammation in these mental disorders occurs without DCD involvement. A significant decrease in serum DCD was found in SCZ patients in remission, possibly explained by the anti-inflammatory effects of antipsychotic therapy.

Collectively, the detected increase in the bloodstream of proteins expressed in the brain may be a consequence of the inflammatory process in BD and SCZ accompanied by increased permeability of the BBB [16].

### Limitations

This study has several limitations. First, the sample sizes of the patient groups were modest, primarily due to the high cost and time-intensive nature of serum proteomic analysis. High-quality proteomic profiling involves the identification of thousands of proteins based on short peptide sequences, and increasing the number of participants substantially raises both labour demands and data processing volumes. Subgroup-specific sample sizes varied across analyses because serum volumes collected from participants varied, and sequential measurements led to a gradual reduction in available sample volume. In addition, the exploratory nature of this study and the lack of a priori sample size calculation should be considered when interpreting negative findings. Based on effect size estimates, this pilot study is sufficiently sensitive to detect strong and moderate effects but may underestimate weak biological signals. Moreover, after adjusting for multiple comparisons, some differences lost significance, indicating only a trend, although the main results remained stable. Therefore, these hypothesis-generating results need to be tested in subsequent studies with larger samples. Second, the sex distribution within the groups was not controlled. However, additional analyses revealed no sex-dependent effects (data not shown). Third, some proteins were undetectable in approximately half of the samples. Therefore, more sensitive assays are needed in future studies. Fourthly, while our results demonstrate the presence of specific proteins and alterations in their levels, we cannot provide a detailed characterisation of their post-translational modifications or tissue origin. Therefore, it remains uncertain whether the observed protein changes directly reflect processes occurring in the brain. Nevertheless, it is widely accepted in biological psychiatry that such changes often correlate. As noted by Belzeaux et al. [76], numerous studies have repeatedly confirmed that brain-expressed proteins essential for nervous system function can be detected in the blood.

## 5. Conclusions

The study identified a complex of changes in serum protein levels in the major mental disorders, namely BD and SCZ. Most of the proteins studied showed significant changes primarily in BD. The main findings include elevated serum NMDAR1 levels in the BD patients with a current depressive episode, decreased ANK3 levels in the same patients and in the SCZ group, and increased ANKRD12 levels in the SCZ patients with predominant negative symptoms and the BD patients with a current depressive episode. In some cases, similar trends were observed in the SCZ patients, but differences may not reach significance due to a high proportion of undetectable values. Collectively, these changes point to glutamatergic dysfunction, impaired neuroplasticity, and increased BBB permeability. Our results suggest that a deficiency of the anchor protein ANK3 may lead to receptor drift into the extrasynaptic space and subsequent entry into the systemic circulation. The detection of high anti-NMDAR antibody titers, particularly pronounced in mixed episodes of BD, indicates possible initiation of a secondary autoimmune cascade. The presence of NMDAR1 fragments in peripheral blood may trigger an immune response that could perpetuate synaptic dysfunction through receptor internalisation. Meanwhile, elevated LRP2 levels may reflect activation of compensatory neuroprotective systems.

Based on our results and published data, we propose the following pathogenetic mechanism. The structural integrity of the glutamatergic synapse is maintained by a complex interaction between extracellular factors and the intracellular scaffold, including SDC2, CASK, SAP97, and ANK3. Reduced ANK3 levels destabilise this process, promoting redistribution of NMDARs into the extrasynaptic space. According to the functional dichotomy model, such receptor shifts can not only initiate neuronal damage but also trigger compensatory induction of neuroprotective systems aimed at stabilising synaptic plasticity. Thus, the studied protein panel supports considering SCZ and BD within a unified synaptopathy concept. In the future, these indicators may serve as markers in comprehensive diagnostic panels. Modulating the SDC2-ANK3-NMDAR axis represents a promising strategy for developing targeted therapies for SCZ and BD. Furthermore, the identified changes in serum protein levels may serve as potential markers for differential diagnosis of clinical forms of these disorders.

## Data Availability

A summary table of proteins identified by panoramic mass spectrometry has been uploaded to the Figshare proteomic data repository and can be found at the following link: https://figshare.com/articles/dataset/Minor_proteins_involved_in_neuronal_functions_in_serum_of_patients_with_major_mental_disorders_according_to_proteomic_analysis/32796687. Additional data are available from the corresponding authors upon reasonable request.

## Abbreviations

ANK3, Ankyrin 3; ANKRD12, ankyrin repeat domain-containing protein 12; BBB, blood-brain barrier; BCB, blood-cerebrospinal fluid barrier; BD, bipolar disorder; DCD, dermcidin; ELISA, enzyme-linked immunosorbent assay; GABA, gamma-aminobutyric acid; HDACs, histone deacetylases; LRP2, low-density lipoprotein receptor-related protein 2; MS, Mass spectrometry; NMDAR, N-methyl-D-aspartate receptor; SCZ, schizophrenia; SIS, synthetic isotope-labelled standard; SRM, selected reaction monitoring; TNRMC, Tomsk National Research Medical Center of the Russian Academy of Sciences.
